# Modeling the Evolution of Major Storm-Disaster-Induced Accidents in the Offshore Oil and Gas Industry

**DOI:** 10.3390/ijerph19127216

**Published:** 2022-06-13

**Authors:** Gaogeng Zhu, Guoming Chen, Jingyu Zhu, Xiangkun Meng, Xinhong Li

**Affiliations:** 1 Centre for Offshore Engineering and Safety Technology (COEST), China University of Petroleum (East China), Qingdao 266580, China; b15040134@s.upc.edu.cn (G.Z.); b18040022@s.upc.edu.cn (J.Z.); 2 Navigation College, Dalian Maritime University, Dalian 116026, China; mxk0117@dlmu.edu.cn; 3 College of Resources Engineering, Xi’an University of Architecture and Technology, Xi’an 710055, China; lixinhong@xauat.edu.cn

**Keywords:** storm disasters, offshore oil and gas accidents, causation evolution, DEMATEL-ISM, complex networks

## Abstract

Storm disasters are the most common cause of accidents in offshore oil and gas industries. To prevent accidents resulting from storms, it is vital to analyze accident propagation and to learn about accident mechanism from previous accidents. In this paper, a novel risk analysis framework is proposed for systematically identifying and analyzing the evolution of accident causes. First, accident causal factors are identified and coded based on grounded theory (GT). Then, decision making trial and evaluation laboratory (DEMATEL) is integrated with interpretative structural modeling (ISM) to establish accident evolution hierarchy. Finally, complex networks (CN) are developed to analyze the evolution process of accidents. Compared to reported works, the contribution is threefold: (1) the demand for expert knowledge and personnel subjective influence are reduced through the data induction of accident cases; (2) the method of establishing influence matrix and interaction matrix is improved according to the accident frequency analysis; (3) a hybrid algorithm that can calculate multiple shortest paths of accident evolution under the same node pair is proposed. This method provides a new idea for step-by-step assessment of the accident evolution process, which weakens the subjectivity of traditional methods and achieves quantitative assessment of the importance of accident evolution nodes. The proposed method is demonstrated and validated by a case study of major offshore oil and gas industry accidents caused by storm disasters. Results show that there are five key nodes and five critical paths in the process of accident evolution. Through targeted prevention and control of these nodes and paths, the average shortest path length of the accident evolution network is increased by 35.19%, and the maximum global efficiency decreases by 20.12%. This indicates that the proposed method has broad applicability and can effectively reduce operational risk, so that it can guide actual offshore oil and gas operations during storm disasters.

## 1. Introduction

Offshore oil and gas production faces a number of harsh environments. In recent years, the number of weather-related natural disasters has grown sharply as have the costs associated with related losses [1,2]. Storm disasters, such as typhoons, hurricanes, tornadoes, and storm surges, significantly impact offshore oil and gas production, and can cause operational shutdown, facility damage, casualties, ecological crises, and even serious social unrest [3,4,5]. During Hurricane Harvey (2017), about 25% of the oil and gas production in the Gulf of Mexico was shut down, and 105 production platforms (15% of the total) were closed, causing a fluctuation in oil and gas prices [6]. To avoid the repeated occurrence of accidents, lessons should be drawn from previous accidents [7,8,9,10,11]. Therefore, it is necessary to analyze the causal factors of major accidents in the offshore oil and gas industry that are caused by storm disasters and explore the accident development process.

To analyze the accident propagation path, it is necessary to carry out an accident investigation and identify causal factors or risk sources. At present, the most commonly used methods for accident analysis in offshore oil and gas operations include RCA, FTA, ETA, barrier analysis, and AcciMap [12]. RCA can identify the root causes of accidents through continuous iteration and improvement [13,14,15]. To ensure the accuracy and integrity of results, RCA requires a certain amount of judgement ability and a relatively exhaustive list of causal factors in advance. FTA is a method for graphically representing the possible causes of top events (i.e., accidents) and their causal relationship through top-down deductive analysis; however, this method cannot consider nonlinear interactions among causal factors [16,17]. ETA is primarily a proactive risk analysis method used for identifying the possible sequence of events after the initial event, but the choice of development paths largely depends on the level of individual knowledge and personal experience [18,19,20]. Through barrier analysis, the risks associated with cascading accidents can be identified to confirm the placement and performance of barriers. Combined with other methods (such as ETA), preventive measures for cascading accidents can be identified for offshore oil and gas operations [21,22]. AcciMap is not only a pure accident investigation tool, but can also capture socio-technical factors and illustrate the interactions among these factors causing corresponding events. With AcciMap, human and organizational factors in offshore oil and gas accidents can be highlighted [23,24,25]. All these methods have advantages but are highly subjective and deductive, thus requiring a strong level of expert knowledge and experience. For the same accident case, researchers with different experience and knowledge levels may obtain completely different results. This is very unfavorable in ensuring the accuracy of identifying the cause factors of accidents.

Complex systems often fail in complex ways, and accidents are usually the result of the interaction of multiple factors. Many methods have been proposed to analyze interactions among factors in accident propagation evaluation, including AHP, ANP, SEM, DEMATEL, and ISM [26,27]. AHP provides a convenient method for multi-objective and multi-criteria decision problems, which derives priority using a nominal scale through paired comparison of elements at the same level. As an extended form of AHP, ANP considers the dependence of elements among different hierarchies; however, the hierarchical structure of both methods is based on subjective expert judgments [28,29,30]. SEM measures the influence of each cause upon the effect, and the regression coefficient can be calculated to express the causal relationships among latent variables (i.e., factors) based on a large number of questionnaires [31,32,33]. By building a structural model containing causal relationships among complex factors, DEMATEL can analyze the interdependences among factors but is limited due to little information [34,35]. ISM is also an effective tool for identifying causal relationships among complex factors, and can help to clarify the hierarchy and priority of factors. Causal relationships are easy to grasp by ISM, but this method requires notable matrix computation resources [36,37]. All the above-mentioned methods have their own application scenarios and functional characteristics. A large number of questionnaires and computing resources are needed to obtain the quantitative interactions among different factors. At the same time, limited information can only judge whether factors are independent. Therefore, a single method cannot achieve efficient and accurate analysis of the interactions among factors in accident propagation evaluation.

To analyze the evolution of accidents, it is necessary to evaluate the propagation path of causative factors. BN has been widely applied for quantitative risk assessment, as forward predictive analysis and backward reasoning diagnosis can be carried out in BN [38,39]. The primary advantage of BN is its probability updating and continuous learning ability. In the field of the offshore oil and gas industry, BN is mainly used to evaluate the dynamic risk evolution of major accidents including blowouts and explosions [40,41], as well as the failure probability and reliability of key equipment and systems; examples are blowout preventer [42], submarine oil and gas pipeline [43,44], drilling riser [45], crude oil separation system [46], and managed pressure drilling system [47,48]. However, it is not easy to obtain the prior probability for BN. Besides, BN focuses on the assessment of the overall risk level of the network, and cannot deal well with the problems of local risk and single path risk. Although BN can determine the importance of nodes through sensitivity analysis, it is hard to study the importance of different paths using the method of path attack. In addition, if the number of causal factors is large and the interaction relationships are complex, there may be a state explosion problem [49].

The limitations of these methods including strong subjectivity, unclear clarification of interactions among factors, and difficulty in assessing path risks. To address issues in the above-mentioned methods, this paper proposes a novel risk analysis framework for systematic identification and evaluation accident propagation. We start with the identification of the accident causes based on GT. Then DEMATEL is integrated with ISM to analyze interactions among factors. Finally, HADY is incorporated into CN to describe the evolution process of accidents and calculate shortest paths. Compared to reported works, the detailed contributions of the proposed method are summarized as follows:

1. GT is developed to objectively identify accident causal factors from original data, which can reduce personnel subjective influences imposed by their knowledge and skill level.
2. The use of DEMATEL-ISM can compensate for the shortcomings of insufficient information and high computing costs. In addition, the way to establish the direct influence matrix is improved according to the interaction matrix obtained from the objective analysis in the previous step.
3. A hybrid algorithm that can calculate multiple shortest paths of the accident evolution under the same node pair is proposed. Combined with the causal factors and interaction relationships obtained by GT and DETAMEL-ISM, CN can quantitatively describe the evolution process of accidents.

## 2. Methodology

To analyze the evolution process of major accidents in the offshore oil and gas industry under storm disasters, a method for the systematic identification and analysis of the evolution process of accident causes is proposed and applied in a case study, which includes four main steps:

1. Identification of accident causal factors.
2. Analysis of the hierarchical structure of accident causes.
3. Research on the evolution process of accident causes.
4. Study of the application in a specific case

**Figure 1:**
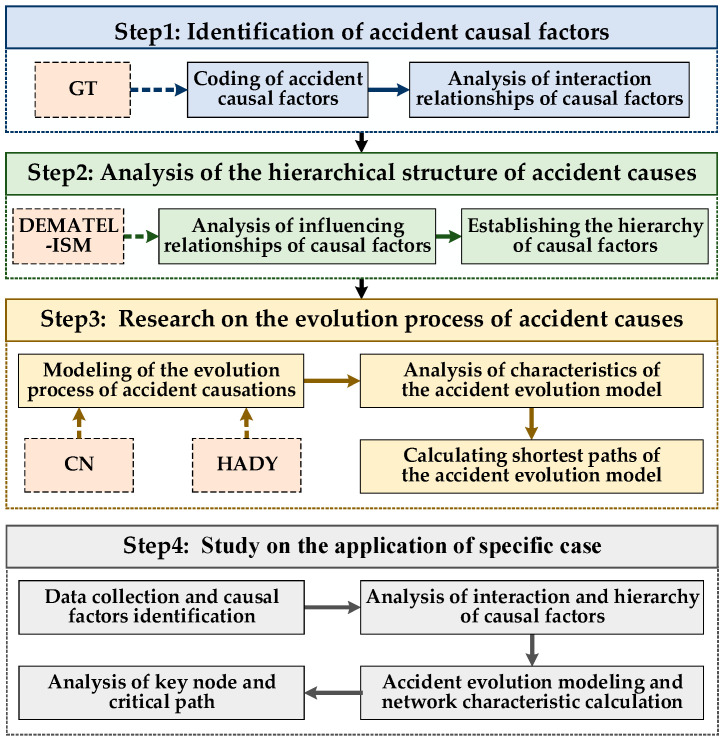
Framework of the proposed methodology.

Firstly, accident causal factors are identified and coded based on GT, and interaction relationships among causal factors are analyzed according to the occurrence frequency of causal factors in the same case. Then, using the DEMATEL-ISM method, the influencing relationships among causal factors are analyzed. When the hierarchy of causal factors is divided, a hierarchy of accident evolution is established. Subsequently, the method of CN is used to determine the evolution model of accident causes, analyze the characteristics of accident evolution, and calculate the shortest paths of accident evolution. Finally, the systematic identification and analysis of the evolution process of accident causes is applied in a case study. The specific process is depicted in Figure 1.

### 2.1. Identification of Accident Causal Factors

As an inductive qualitative research method rooted in original data, GT is an effective tool to identify accident causal factors. So far, there have been three schools: classic GT [50], programmatic GT [51] and constructivist GT [52]. As an objective induction method, the obtained analysis results are more objective and reliable as they are based on accident-related data [53]. Considering the standardization and simplicity of programmed language and in line with the method, this paper is based on programmatic GT. The coding process of programmatic GT includes three main steps: open coding, axial coding, and selective coding [54]. The method of GT emphasizes constant comparison and abstraction to form a reliable theory; thus, theoretical saturation tests should be performed after coding.

### 2.2. Analysis of the Hierarchical Structure of Accident Causes

To analyze complex interaction relationships among accident causes, a suitable and efficient analysis method is needed. As important tools for system analysis and decision making, DEMATEL [55,56] and ISM [57] have been widely used in various fields. Nevertheless, DEMATEL fails to consider the influence of factors on itself, while ISM fails to consider the strength of influence relationships among factors. The integrated DEMATEL-ISM method compensates for the deficiencies of both tools, and the integrated method also reduces the difficulty for effectively calculating the reachability matrix in ISM. By setting the threshold, DEMATEL-ISM eliminates the weak influencing relationship in the system and simplifies the structure of the system. 

In this paper, the traditional method of DEMATEL-ISM is improved, by incorporating the following points:

1. When constructing the index system of causal factors, the categories obtained from GT are absorbed, and are put into the set *Y* (*y_i_&y_j_* ∈ *Y*, *i* = 1, 2, …, *n*; *j* = 1, 2, …, *n*) of causal factors, to reduce the influence of subjectivity and avoid the inconsistency of the index scope.
2. When establishing the matrix ***F***, the matrix ***R*** is referred based on expert scoring, to obtain more objective analysis results.
3. In the evolutionary hierarchy of accidents obtained from ISM analysis, the category frequency and the strength of influence relationships among factors obtained from GT and DEMATEL are presented simultaneously.

The specific algorithm steps are as follows:

1. Establish the matrix ***F*** = [*f_ij_*]*_n_*_×*n*_, where *f_ij_* represents the direct influence of factor *y_i_* on factor *y_j_* (*y_i_&y_j_* ∈ *Y*, *i* = 1, 2,…, *n*; *j* = 1, 2,…, *n. Y* is a set of system factors.). {0, 1, 2, 3} respectively represent {no influence, weak influence, medium influence, strong influence} in the matrix ***F***.
2. Normalize the matrix ***F*** and obtain the matrix ***C*** = [*c_ij_*]*_n_*_×*n*_, where *c_ij_* ∈ [0, 1]:

(1) $$C=\frac{1}{\underset{1\leq i\leq n}{\max}\sum_{j=1}^{n}y_{ij}}F$$

3. Develop the matrix ***T*** = [*t_ij_*]*_n_*_×*n*_ which is established to couple the direct and indirect influence relationships among factors:

(2) $$T=\underset{k\to \infty }{\lim}(C+C^{2}+\ldots +C^{k})=C{\left(I-C\right)}^{-}{}^{1}$$

4. Calculate the *b_i_*, *d_i_*, *c_i_* and *a_i_* of each factor:

(3) $$b_{i}=\sum_{j=1}^{n}t_{ij},d_{i}=\sum_{j=1}^{n}t_{ji},c_{i}=b_{i}+d_{i},a_{i}=b_{i}-d_{i}$$

5. Develop the matrix ***H*** = [*h_ij_*]*_n_*_×*n*_ mainly for the purpose of considering the influence of factors on itself:

(4) H=T+I

6. Develop the matrix ***K*** = [*k_ij_*]*_n_*_×*n*_, which is established to simplify the weak influence relationship between factors and highlight the hierarchy of the system by selecting an appropriate threshold:

(5) $$\{\begin{array}{c} k_{ij}=1,\;\;t_{ij}\geq \lambda \\ k_{ij}=0,\;\;t_{ij}<\;\lambda \end{array}$$

7. Calculate the *Q_i_* and *P_i_*:

(6) $$\begin{array}{c} Q_{i}=\left\{y_{i}|y_{i}\in Y,\;\;k_{ji}=1\right\}\\ P_{i}=\left\{y_{i}|y_{i}\in Y,\;\;k_{ij}=1\right\} \end{array}$$

8. Establish the condition for hierarchical partitioning:

(7) $$P_{i}\cap Q_{i}=P_{i}$$

If the above condition is satisfied, it is proved that the corresponding factors in *P_i_* can all be found in *Q_i_*. Therefore, these factors are at a higher level. At the same time, the corresponding row *i* and column *j* are deleted from the matrix ***K***, and then step 7 and step 8 are repeated until all factors are deleted. The hierarchical structure of all factors is determined according to the order in which the factors are deleted.

### 2.3. Research on the Evolution Process of Accident Causes

Based on graph theory and statistical mechanics, CN is an important tool for analyzing the structural characteristics and development process of complex systems. CN can describe the process of risk evolution and effectively evaluate dependent relationships among different factors. Many complex systems or processes in nature can be described by CN [58]. CN has been widely used in a variety of fields, such as transportation networks [59,60], power systems [61,62], natural disasters [63], and offshore oil and gas production [49,58].

According to the organization form of network nodes, the four basic models of regular networks, random networks, small-world networks, and scale-free networks can be classified. The small-world network is characterized by a large clustering coefficient and small average path length. The scale-free network reflects node growth and preference dependence based on small-world network characteristics, which are closer to the characteristics of network models in the real world [64,65].

#### 2.3.1. Modeling of CN

The CN graph is a data structure composed of nodes, edges, and weights. The CN graph is usually represented mathematically by *G* = (*V*, *E*, *W*). The node in *V* is denoted as *v_i_* by its order *i*. The edge linking *v_i_* with *v_j_* in *E* is denoted as *e_ij_*. If *e_ij_* and *e_ji_* represent the same edge, the graph is an undirected graph; otherwise, it is a directed graph. Each element in *W* expresses the weight of *e_ij_*, which is denoted as *w_ij_*. If each *w_ij_* is equal to 1, the graph is an unweighted graph; otherwise, it is a weighted graph. In this paper, the graph is a weighted network graph. The principal assumptions for the modelling can be summarized as follows: different events in the process of accident evolution can be represented by nodes, and the associations among events can be represented by weighted edges. Causal factors (nodes) are obtained by GT, and influence relationships (weighted edges) among factors obtained by DEMATEL-ISM. The accident evolution model is constructed by CN.

#### 2.3.2. Characterization of CN

Every complex network has its unique topological structure and connectivity, and the transmission process of internal information cannot be obtained only by means of observation. Therefore, we need the specific measurement method to evaluate the characteristics of the complex network, so that we can have a deeper understanding of it. Through the discrimination and analysis of network characteristics, the critical nodes, critical paths, propagation modes, and development trends of accident evolution can be obtained. These findings can provide references for accident prevention, control and emergency. The principal models and the presentation of the main measurements for complex network characterization [49,64,65] are as follows:

The *k_i_* of *v_i_* refers to the number of edges connected to the node in the network. In a directed network, the *k_i_* is composed of two components: the $k_{i}^{\mathrm{out}}$, which is equal to the number of outgoing edges or successor nodes, and the $k_{i}^{\mathrm{in}}$, which is equal to the number of incoming edges or predecessor nodes. The *k_i_* is the simplest and most effective concept for measuring the importance of a node in a network:

(8) $$k_{i}^{\mathrm{out}}=\sum_{j}e_{ij},k_{i}^{\mathrm{in}}=\sum_{j}e_{ji},k_{i}=k_{i}^{\mathrm{out}}+k_{i}^{\mathrm{in}}$$

where *e_ij_* is a connected edge from *v_i_* to *v_j_*. If this edge exists, *e_ij_* = 1; otherwise, *e_ij_* = 0. Similarly, *e_ji_* is a connected edge from *v_j_* to *v_i_*, and if this edge exists, then *e_ji_* = 1; otherwise, *e_ji_* = 0.

*P*(*k*) is defined as the probability of selecting a node randomly in the network whose degree is *k*, i.e., the ratio of the number of nodes with *k_i_* = *k* to the number of all nodes in the network. *P*(*k*) describes the most basic topological characteristics of the network and is also an important scale used to identify network types:

(9) $$P(k)=\frac{n_{k}}{n}$$

where *n_k_* is the number of nodes in the network with the degree *k*, and *n* is the order of the adjacency matrix (i.e., the number of nodes in the network).

*CC_i_* represents the connection relationship among all nodes that are adjacent to a node in the network. *CC_i_* is defined as the proportion of the actual number of connected edges of these neighboring nodes to the maximum number of possible connected edges. *CC_i_* reflects the importance and subsequent growth of this node among neighboring nodes. *CC* is defined as the mean value of the *CC_i_* of all nodes in the network, which is an important index to measure network collectivization and represent the clustering ability of the network:

(10) $$CC_{i}=\frac{e_{i}}{m_{i}(m_{i}-1)},CC=\frac{1}{n}\sum_{i=1}^{n}CC_{i}$$

where *e_i_* is the actual number of connected edges of neighboring nodes for *v_i_*, and the interconnection between two nodes in a directed network serves as two edges. *m_i_* is the number of neighboring nodes of *v_i_*, which is defined as the sum of successor and predecessor nodes in a directed network (repeated nodes are only counted once).

*BC_i_* represents the probability of the shortest paths through this node in the network, and it is defined as the proportion of the number of shortest paths through this node to all shortest paths. *BC_i_* reflects the load and influence of a particular node in the network, and network robustness can be assessed by attacking nodes with high *BC_i_*:

(11) $$BC_{i}=\sum_{s\neq i\neq t}\frac{n_{st}(i)}{N_{st}}$$

where *n_st_* is the number of shortest paths from *v_s_* to *v_t_* that pass through *v_i_*, and *N_st_* is the total number of shortest paths from *v_s_* to *v_t_*.

The degrees of dispersion of *BC_i_* for different nodes are large, thus normalization processing is performed:

(12) $$BC_{i}^{\prime }=\frac{BC_{i}}{\left(n-1)(n-2\right)}$$

*l_ij_* is defined as the sum of edge weights for the shortest path between *v_i_* and *v_j_* (a pair of nodes) in the network. *l_ij_* is used to measure the shortest distance between two nodes in a network, which, in this paper, represents the maximum connectivity between two nodes. The maximum value of *l_ij_* between any two nodes *v_i_* and *v_j_* is denoted as *D* of the whole network. *L* reflects the overall connectivity and connection efficiency of the network, which is of great significance. *L* is defined as the mean value of *l_ij_* between two random connected nodes in a directed network:

(13) $$L=\frac{1}{n_{l}}\sum_{i\neq j}l_{ij}$$

where *n_l_* is the number of connected node pairs in the network.

To avoid divergence in the calculation of *L* caused by unconnected node pairs, the concept of global efficiency is proposed. *GE* is negatively correlated with *L*, which can quantify the efficiency of the network in transmitting information between nodes. *GE* is an important index to measure the connectivity of one network, and is defined as the mean value of the reciprocal of *l_ij_* between two random nodes in the network:

(14) $$GE=\frac{1}{n(n-1)}\sum_{i\neq j}\frac{1}{l_{ij}}$$

If there is an interconnected path between *v_i_* and *v_j_* in the directed network, both nodes are strongly connected. If every two nodes in a network are strongly connected, this directed network graph is a strongly connected graph. The subgraph of a directed network graph with the maximum connection strength is called the strongly connected component, and the strongly connected component with the largest scale is called the maximum strongly connected component. *MK* is defined as the number of nodes in the maximum strongly connected component of the directed network graph, which reflects the robustness of the network under attack.

As the sum of edge weights is needed to calculate the shortest path, the concept of entropy is used to represent edge weights in the network, to meet the needs of studying the network characteristics and searching paths:

(15) $$ew_{ij}=\ln(10^{4-w_{ij}})$$

#### 2.3.3. Shortest Paths of CN

There are two commonly used algorithms for shortest paths in CN: the Floyd algorithm and the Dijkstra algorithm. The Floyd algorithm [66] adopts the idea of dynamic programming, and it is suitable for solving the shortest path problem between any two nodes. The Dijkstra algorithm [67] adopts a greedy search strategy, and it is suitable for solving the shortest path problem with a single source. The Yen algorithm [68] is currently the most widely used algorithm to solve the problem of K shortest paths. Considering the lower time complexity of the Dijkstra algorithm, the shortest path is calculated by the Dijkstra algorithm. However, the conventional Dijkstra algorithm can only calculate one shortest path for the same initial node and target node. Therefore, the HADY is proposed to calculate all shortest paths of specified node pairs in the network.

**Figure 2:**
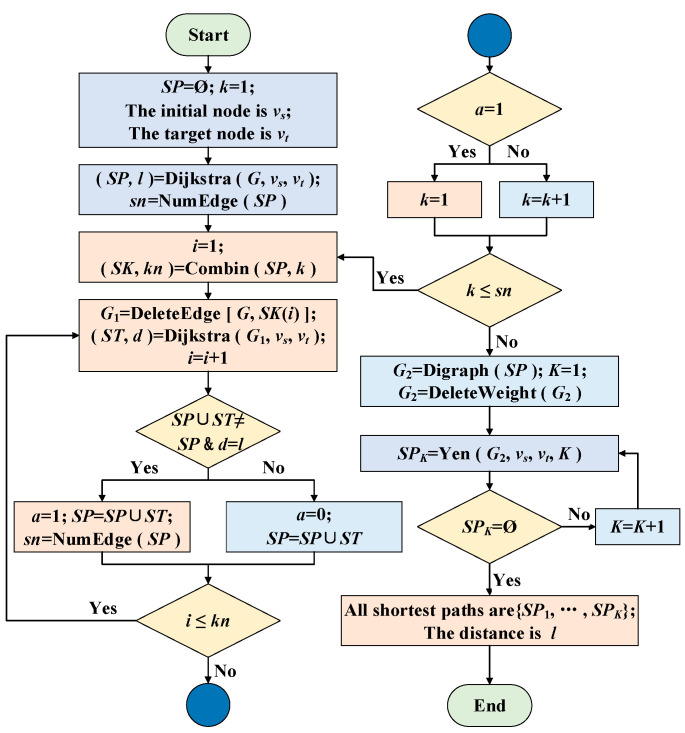
Flow chart of HADY.

The idea of HADY is firstly to obtain a shortest path of the specified node pair in the original network by the Dijkstra algorithm. By constantly removing the edges and their combinations of known shortest paths from the original network, the shortest path network of the specified node pair is obtained, and then, the shortest path network is transformed into an unweighted network. Finally, the Yen algorithm is used to solve K shortest paths of the shortest path network, and multiple shortest paths, sorted by the number of nodes, are obtained. The flow chart of HADY is shown in Figure 2.

The detailed steps of this algorithm are as follows:

1. Set the edge set of shortest paths *SP* = Ø (the initial *SP* is the empty set). The initial node is *v_s_*, and the target node is *v_t_*. The initial *k* is 1.
2. The Dijkstra algorithm is used to obtain a shortest path from *v_s_* to *v_t_* in the original network *G*. All edges of the shortest path are put into the set *SP*. The number of edges is denoted as *sn*, and the length of the path is denoted as *l*.
3. List all combinations of *k* edges taken from the set *SP* and put them into the set *SK*. There are *kn* combinations in total, and the initial *i* is 1.
4. Take the *i*-th combination from *SK* and delete the set of corresponding edges *SK*(*i*) in the original network *G*. The Dijkstra algorithm is used to find the shortest path from *v_s_* to *v_t_* in the modified network *G*_1_, and all edges of the shortest path are put into the set *ST*. The length of the path is denoted as *d*, and *i* = *i* + 1.
5. Determine whether a new edge of the shortest path is added. If *d = l* and *SP* ∪ *ST* ≠ *SP*, a new edge has been added. Then, the logical value *a* = 1, *SP* = *SP* ∪ *ST*, and the number of edges is recalculated and denoted as *sn*. Otherwise, no new edge is added, the logical value *a* = 0, *SP* = *SP*, and the number of edges is denoted as *sn*.
6. Determine whether all combinations have been taken out. If *i* ≤ *kn*, not all combinations are taken out, and step 4 will be taken. Otherwise, if all combinations have been taken out, step 7 will be taken.
7. Determine whether this combination should be carried out again. If *a* = 1, this combination should be carried out again, then *k* = 1. Otherwise, there is no need to carry out this combination again, then *k* = *k* + 1.
8. Determine whether all combinations have been listed. If *k* ≤ *sn*, not all combinations have been listed, then step 3 will be taken. Otherwise, all combinations have been listed, then step 9 will be taken.
9. The set of all edges of shortest paths *SP* is used to form the shortest path network graph *G*_2_. All edge weights in *G*_2_ are set to 1 (i.e., unweighted network), and *K* = 1.
10. *K* shortest paths from *v_s_* to *v_t_* in the unweighted shortest path network graph *G*_2_ are obtained by the Yen algorithm and put into the set *SP_K_*.
11. Determine whether the combined algorithm has been completed. If *SP_K_* = Ø, step 12 will be taken; otherwise, *K* = *K* + 1 and step 10 will been taken.
12. The sets *SP*_1_ to *SP_K_* are all the shortest paths from *v_s_* to *v_t_* in the original network *G*, and the length of the shortest path is *l*.

## 3. Case Study

**Figure 3:**
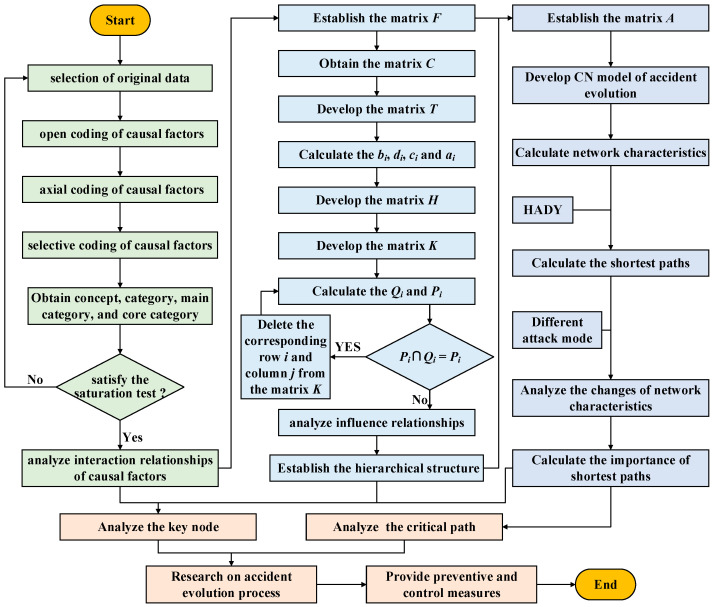
Flow chart of the case study.

A case study of major offshore oil and gas industry accidents caused by storm disasters is carried out, applying the proposed method. Firstly, causal factors are identified. Then, the hierarchy structure of the evolution of causes is studied. Finally, the evolution model of the accident causes is constructed to analyze the network characteristics and calculate the shortest path. The flow chart of the case study is shown in Figure 3.

The selection of original data is the first step of the research, and its accuracy and detail are the foundation of successful research, which also guarantees the reliability of the results. To guarantee the accuracy and reliability of the results, the data selected in this paper are mainly obtained from official accident investigation reports, accident announcements and accident statistics of competent authorities, news reports of mainstream media, and accident databases of authoritative institutions. Meanwhile, we only selected accidents with detailed processes, and we focused on those cases that cause serious structural damage or casualties. In addition, three groups of experts (from university, research institute, and offshore platform) were responsible for the analysis. Each team consisted of three members and performed an independent analysis of the same data. Through comparison and analysis, the final results were extracted to avoid inconsistency of concepts and categories caused by differences in theoretical sensitivity. Through these measures, the accuracy, reliability, and availability of the results are further guaranteed.

### 3.1. Identifying Causal Factors of Offshore Storm Accidents

#### 3.1.1. Coding of Accident Causal Factors

In this paper, a total of 78 major accidents in the offshore oil and gas industry caused by storms were selected, involving 215 different types of data. Details of the accidents are presented in Appendix A [69,70,71,72,73,74]. Among the selected 78 cases, 72 cases were randomly selected for coding, and the remaining six cases were used for theoretical saturation tests. After coding of the accident-causing factors based on the selected accident cases, the results of three groups of analysis were compared, discussed, and integrated. Subsequently, a total of 63 concepts, 25 categories, nine main categories, and three core categories were obtained, as shown in Table 1. Finally, an experienced researcher was invited to conduct independent coding of the remaining six cases (No.5, No.12, No.18, No.31, No.42, and No.66) based on the same method. It was found that no new coding was generated, proving that the existing coding has covered all the accident cases. This indicated that the results satisfy the theoretical saturation test requirements. If the new coding appears, this indicates that the existing coding does not cover all accident cases. Then more accident cases need to be added and recoded until the verification test is passed. This step can further ensure the accuracy and reliability of the analysis results.

According to Table 1, inclement weather (*y*_1_), loss of watertight integrity (*y*_13_), lack of risk awareness (*y*_21_), poor state of the platform (*y*_14_), damage of support structures (*y*_10_), damage of platform facilities (*y*_12_), and damage of watertight structures (*y*_11_) are the most frequently occurring categories. Attention needs to be focused in those categories.

#### 3.1.2. Analyzing Interaction Relationships among Causal Factors

To analyze interaction relationships among different categories, the matrix ***S*** = [*s_ij_*]*_n_*_×*n*_ is defined, where *s_ij_* (*i* = 1, 2, …, *n*; *j* = 1, 2, …, *n*) represents the interaction value between the two categories. *s_ij_* is defined as the ratio of the number of intersection cases to the number of union cases the two categories belong to. After calculating and processing, the matrix ***R*** = [*r_ij_*]*_n_*_×*n*_ is obtained. {0, 1, 2, 3} in the matrix represents {no interaction, weak interaction, medium interaction, strong interaction}, respectively. The calculation rules are as follows:

(16) $$\left\{ \begin{array}{l} r_{ij}=0,\;\;\;\;\;\;\;s_{ij}=0\\ r_{ij}=1,\;\;\;\;\;0<s_{ij}\leq 0.1\\ r_{ij}=2,\;\;0.1<s_{ij}\leq 0.4\\ r_{ij}=3,\;\;\;\;\;\;\;s_{ij}>0.4 \end{array}\right.$$

### 3.2. Developing the Hierarchy of Causes of Offshore Storm Accidents

Based on the 25 causal factors (i.e., the categories) extracted by GT, DEMATLE-ISM is used to analyze influencing relationships and establish the hierarchical structure of the accident causation evolution.

#### 3.2.1. Analyzing Influence Relationships among Causal Factors

**Figure 4:**
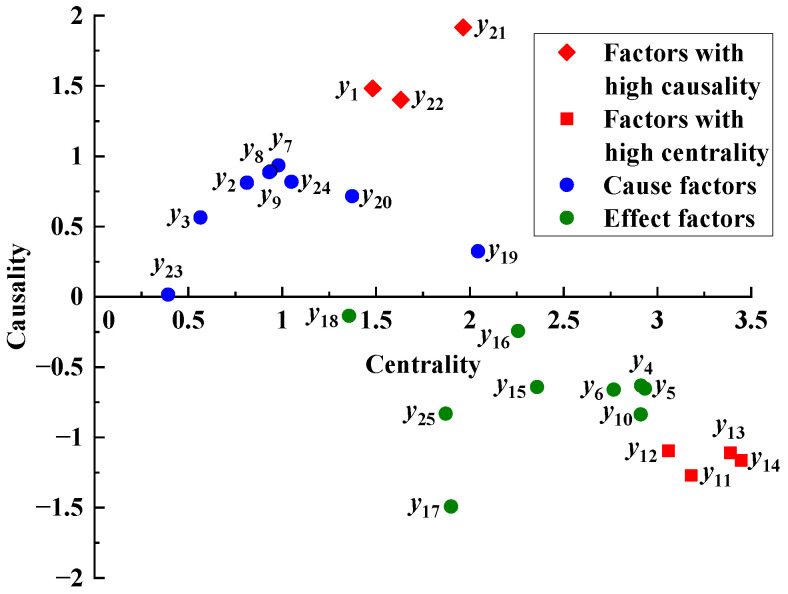
Centrality and causality of factors.

The centrality and causality of accident-causing factors are plotted on a Cartesian coordinate system, as shown in Figure 4.

According to Figure 4, factors such as damage to watertight structure (*y*_11_), damage to platform facility (*y*_12_), loss of water-tightness (*y*_13_), and poor state of the platform (*y*_14_) have higher centrality. This indicates that these factors play an important role in the accident-causing process. Inclement weather (*y*_1_), lack of risk awareness (*y*_21_), and inadequate safety training (*y*_22_) have higher causality, indicating that these factors greatly impact other factors in the process of accident development and are the main causes of accidents.

#### 3.2.2. Establishing the Hierarchical Structure of Causal Factors

**Figure 5:**
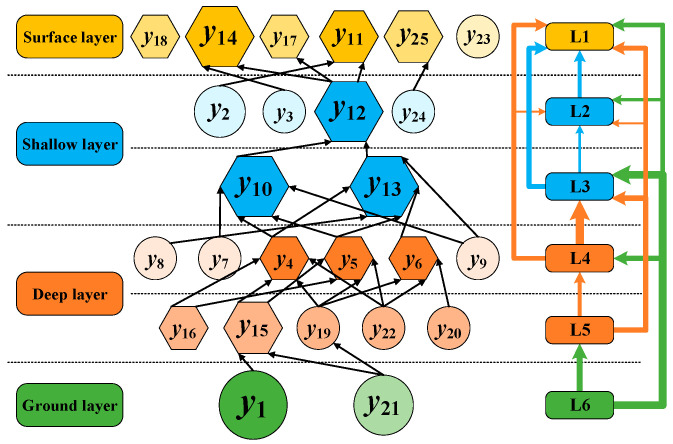
Hierarchical structure of causal factors.

In the transformation process from the matrix ***H*** to the matrix ***K***, *λ* = 0.09 is chosen as the appropriate threshold. According to the obtained ***K***, the system hierarchy is divided, and then, the accident evolution hierarchy is determined. The result is shown in Figure 5, in which only the influencing relationships among factors at adjacent levels are presented. In Figure 5, the circle shape represents the cause factor, while the hexagon shape represents the result factor. The size represents the category frequency obtained in the analysis of GT, which is divided into four levels (the larger the size, the higher the frequency of occurrence). The shading represents the centrality of the factor, which is divided into three levels (the darker the color, the greater the centrality). The thickness of the line between different levels represents the influence relationship between levels (the thicker the line, the stronger the influencing relationship).

According to Figure 5, the 25 causal factors are divided into six layers. L1 is the surface layer, which is the direct cause of the accident. L2 and L3 form the shallow layer, and represent early signs of the accident. L4 and L5 form the deep layer, and represent the concentrated emergence of accident causes. L6 is the ground layer, which is the root of the accident. In terms of factors, inclement weather (*y*_1_) and lack of risk awareness (*y*_21_) are the root causes of accidents. Collision of fixed object at sea (*y*_4_), collision of floating object at sea (*y*_5_), and collision of object on the platform (*y*_6_) have complicated influence relationships with other factors that belong to upper and lower levels. Damage to support structure (*y*_10_) and loss of water-tightness (*y*_13_) have high centrality and occurrence frequency, and are key factors in accidents. Damage to platform facility (*y*_12_) is the only path for the propagation of the causative factors at upper and lower layers. When the watertight structure is damaged (*y*_11_) and the platform is in poor condition (*y*_14_), accidents are more likely to happen than usual. At this time, all necessary control measures should be taken, and emergency work should be carried out to mitigate the consequences of the accident.

### 3.3. Analyzing the Evolution Process of Offshore Storm Accidents

Based on the modeling principle of CN, the accident evolution model of the offshore oil and gas industry under storm disasters is established, combining 63 concepts extracted from GT by analyzing the evolution hierarchy of accidents. Using this model, the accident evolution characteristics are analyzed, and the shortest evolution paths are calculated.

#### 3.3.1. Modeling the Evolution Process of Accident Causes

**Figure 6:**
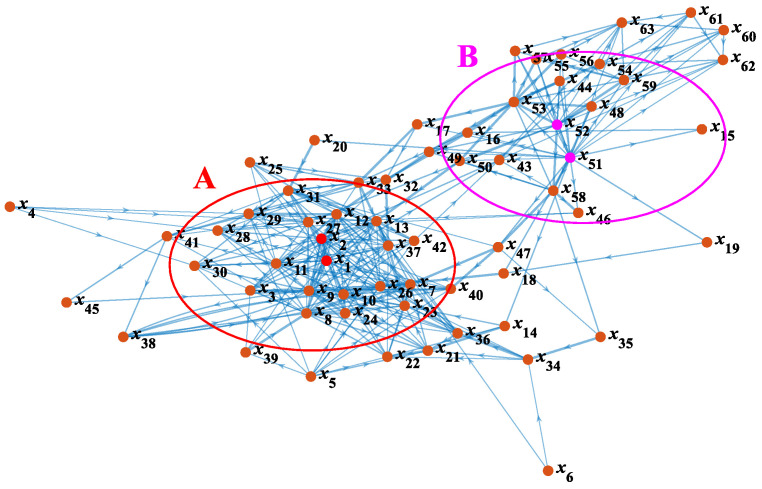
CN model of accident evolution.

In line with the construction method of the matrix ***R***, the matrix ***RX*** is established. In combination with the matrix ***F***, 63 concepts are put into the set of *X* (*x_i_&x_j_* ∈ *X*) to establish the matrix ***A*** = [*a_ij_*]*_n_*_×*n*_, where *n* represents the order of ***A***, and *a_ij_* represents the connectivity from *x_i_* to *x_j_*. In the matrix ***A***, {0, 1, 2, 3} represents {no connectivity, weak connectivity, medium connectivity, strong connectivity}, respectively. According to the matrix ***A***, a weighted directed CN graph is plotted, in which *v_i_* is represented by *x_i_*, and *a_ij_* is taken as *w_ij_*. In the established graph, the thickness and length of the line represent the connectivity between two nodes. Corresponding to thicker and shorter lines, a larger weight represents a greater connectivity, as shown in Figure 6.

In Figure 6, the whole network is obviously divided into two subnetworks, which are the region A centered on *x*_1_ (strong ocean storm) and huge *x*_2_ (ocean wave), and the region B centered on *x*_51_ (inadequate risk perception) and *x*_52_ (inadequate risk response). The internal connection of these two subnetworks is complex and the average connectivity is high, which means that internal factors are closely related. The region A and the region B are connected through intermediate nodes including lack of *x*_17_ (watertight subdivision isolation), *x*_47_ (violation of towing operation), et al. The nodes at the edge of the subnetwork have higher connectivity with intermediate nodes, but the internal nodes of the two subnetworks need to be connected through edge nodes as medium. The average connected path between these nodes is long and the connection relationship between them is sparse. The whole network shows clear community structure characteristics.

#### 3.3.2. Analyzing the Evolution Characteristic of Accident Causes

The *k_i_*, $k_{i}^{\mathrm{in}}$, and $k_{i}^{\mathrm{out}}$ in the network are calculated, and nodes with high values are: *x*_1_ (strong ocean storm), *x*_2_ (huge ocean wave), *x*_8_ (collision of buildings), *x*_9_ (collision of vessels), *x*_12_ (collision of cargos), *x*_13_ (collision of lifeboats), *x*_37_ (list of the platform) and *x*_51_ (inadequate risk perception). At the same time, *P*(*k*) in the network is calculated, which presents the characteristics of power law distribution (*P*(*k*) ~ *k^−β^*) where *β* = 2.82, which is consistent with the distribution feature of *β* = 2~3 in a scale-free network. The *CC_i_* and *BC_i_* of all nodes in the network are calculated, as shown in Table 2.

**Figure 7:**
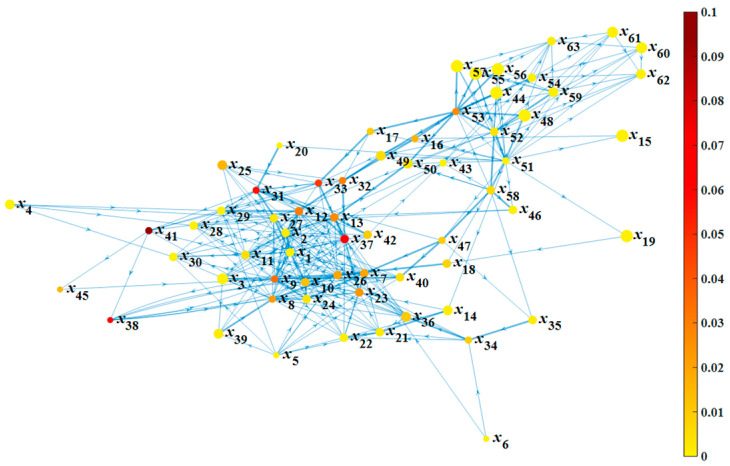
Characteristic of the evolution network of accidents.

According to Table 2 and based on Figure 6, the network characteristic graph of the accident evolution is drawn, as shown in Figure 7. The size of the node represents the value of *CC_i_*, and the color shade of the node represents the value of $BC_{i}^{\prime }$. A larger size represents greater *CC_i_*, and a darker shade represents greater $BC_{i}^{\prime }$.

In Table 2 and Figure 7, the maximum *CC_i_* is 0.5, and the corresponding nodes are *x*_15_ (improper screws), *x*_19_ (design defect of single hull tankers), *x*_44_ (poor performance of protective clothing), and *x*_48_ (wrong wellhead connection). However, the number of neighboring nodes of these nodes is too small to be representative. Among the other nodes, the maximum *CC_i_* is 0.45, corresponding to *x*_55_ (insufficient auxiliary vessels), *x*_56_ (insufficient lifeboats), and *x*_57_ (insufficient emergency protective equipment), and these nodes have many neighboring nodes, indicating their relatively high collectivization. Therefore, adequate emergency equipment can effectively suspend the rapid escalation of accidents. The *CC* of the network is 0.223, which is far less than 1, but also much more than 1/*n* = 0.016. This is in line with the characteristics of the real-world network, which proves the feasibility of the established network. 

In Table 2 and Figure 7, nodes with high *BC_i_* are *x*_41_ (power system failure), *x*_38_ (thruster failure), *x*_37_ (list of the platform), *x*_31_ (flooding of deck), and *x*_33_ (flooding of subdivision), indicating that there are multiple shortest paths through these nodes. In other words, these nodes are where the accident evolution most likely passes. Therefore, the proper functioning of the power system, thrusters, and other equipment should be ensured, as this can effectively reduce the network connectivity and the possibility of accidents. The same applies to the stability and water tightness of the platform.

The *L* of the network is 12.69, and the *D* of the network is 32.24. *L* is far less than *D*, indicating that the accident evolution has small-world network characteristics. The *GE* is 4.01 × 10^−2^, which indicates that the network has strong connectivity ability and efficiency. The *MK* is 21, indicating that interconnected paths exist among these 21 nodes in the network, and the overall robustness of the network is strong.

#### 3.3.3. Calculating Shortest Evolution Paths of Accident Causations

**Figure 8:**
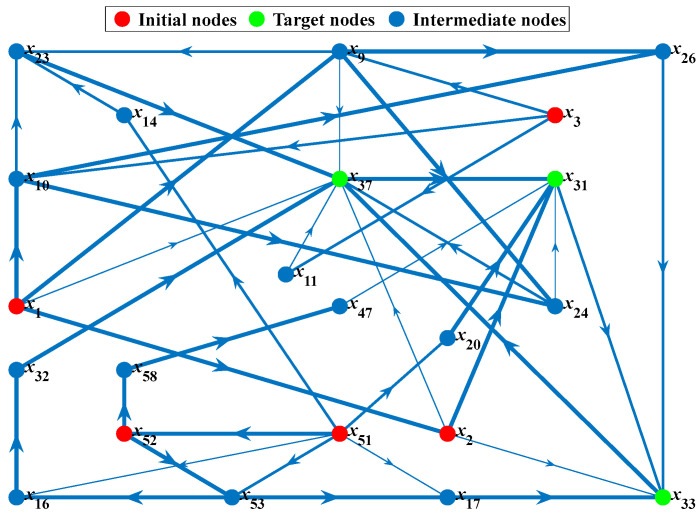
Network diagram of shortest paths.

According to the evolution hierarchical structure and statistical frequency shown in Figure 5 and Table 1, *x*_1_ (strong ocean storm), *x*_2_ (huge ocean wave), *x*_3_ (rapid ocean current), *x*_51_ (inadequate risk perception), and *x*_52_ (inadequate risk response) in the ground layer (L6) are selected as initial nodes, and *x*_31_ (flooding of decks), *x*_33_ (flooding of cabins), and *x*_37_ (list of the platform) in the surface layer (L1) are selected as target nodes. These nodes result in a total of 15 node pairs (i.e., 15 shortest path groups). The proposed HADY is used to obtain the shortest path network graphs, and the thickness of the line represents the connectivity between two nodes, where stronger connectivity corresponds to thicker lines, as shown in Figure 8. The network graphs of shortest paths for different target nodes are presented in Appendix B.

According to the HADY, the shortest paths and lengths are calculated, and a total of 43 shortest paths are obtained. The specific results are shown in Table 3.

In Table 3, a total of five pairs of nodes have the least number of paths (only one path), and the node pair with the largest number of paths is path_3, which has eight shortest paths. For the same node pair, the more paths there are, the more difficult it is to control the evolution process. Because even if one of the paths (i.e., the causative chain) is blocked, there may be other evolution paths. Therefore, attention should be paid to node pairs with multiple shortest paths, and the node or edge with the highest blocking efficiency should be selected as control. For example, if the edge *x*_37_ → *x*_31_ or the node *x*_37_ is removed from the eight shortest paths of path_3, the number of shortest paths decreases from eight to two, and the blocking efficiency increases to 75%.

In addition, the node pair of the minimum shortest path length is (*x*_2_, *x*_31_) with a length of 2.30, while the node pair of the maximum shortest path length is (*x*_3_, *x*_31_) with a length of 13.82. The shorter path length represents stronger connectivity and greater possibility of accident occurrence. All shortest paths have at least one edge and five edges at most. For the same path length, more edges represent more nodes, i.e., the development process of accidents involves more causative factors. This also indicates that there are more optional control measures.

In general, the longer the shortest path length of a node pair, the more paths and the more edges there are. However, high risk features (e.g., shorter path length, fewer edges, and more paths) generally do not appear on the same path. Therefore, when controlling the evolution path of accidents, the high risk features of paths should be identified first. Subsequently, appropriate prevention and control measures should be taken.

To quantify the importance of different shortest path groups, according to the 15 shortest path groups listed in Table 3, intentional attacks of two modes are carried out on the original network:

1. Attack only the edges of the shortest path;
2. Attack both the edges and intermediate nodes of the shortest path.

**Figure 9:**
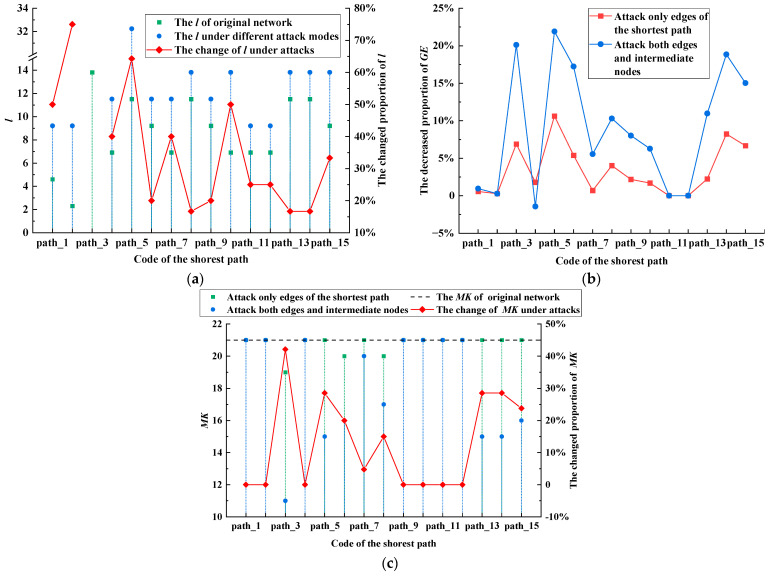
Comparison of (**a**) *l*, (**b**) *GE* and (**c**) *MK* under different attack modes.

The *l*, *GE* and *MK* under the two attack modes are analyzed, as shown in Figure 9.

In Figure 9a, the *l* of different shortest path groups under the attack mode 1 is consistent with that under the attack mode 2. Compared with the original network, all *l* increase under both attack modes. The average increased proportion of all *l* is 35.19%, indicating that the shortest path length increases and the connectivity reduces under both attack modes, which can reduce the possibility of accidents. In Figure 9b, the average decreased proportion of *GE* is 3.43% under the attack mode 1, and the average decreased proportion of *GE* is 8.94% under the attack mode 2. A comparison of both attack modes shows that the change trend of *GE* is almost identical. In Figure 9c, the average *MK* of the network is 20.73 under the attack mode 1, and the average *MK* of the network is 18.1 under the attack mode 2. Compared with the attack mode 1, the *MK* of the network decreases most when attacking path_3 under the attack mode 2, and the decrease proportion is 42.11%. Compared with the original network, the attack effects of the two attack modes are not ideal. This indicates that the original network has strong robustness. In such a case, attacking a single path or node can hardly reduce the robustness of the network.

The research above treats multiple shortest paths of the same node pair as a whole. To study the influences of different shortest paths of the same node pair on the network, it is necessary to integrate various network characteristics under attack. After normalization and summation, the importance of different shortest paths *PS* for the same node pair is obtained. The calculation formula is as follows:

(17) $$PS=\frac{GE-GE_{\min}}{GE_{\max}-GE_{\min}}+\frac{MK-MK_{\min}}{MK_{\max}-MK_{\min}}+\frac{MS-MS_{\min}}{MS_{\max}-MS_{\min}}$$

where *GE* and *MK* are the values after attacking the shortest path. *MS* is the importance of intermediate nodes for the shortest path. *GE*_max_, *MK*_max_, and *MS*_max_ are the maximum values of all shortest paths for the same node pair. *GE*_min_, *MK*_min_, and *MS*_min_ are the minimum values of all shortest paths for the same node pair.

According to Equation (17), the importance of different shortest paths for the same node pair are calculated and sorted in the same node pair, as shown in Table 4.

### 3.4. Results of the Case Study

According to the above analysis results, the shortest path groups that need to be focused on are as follows:

1. The path_3 (*x*_3_, *x*_31_), which is the shortest path group hardest to prevent, i.e., the one with the most paths. There are eight shortest paths, and each path contains the initial node *x*_3_ (rapid ocean current) and the target node *x*_31_ (flooding of decks). The intermediate nodes are *x*_9_ (collision of vessels), *x*_10_ (collision of debris), *x*_11_ (collision of cantilever deck), *x*_23_ (fracture of legs), *x*_24_ (collision of vessels) and *x*_37_ (list of the platform).
2. The path_2 (*x*_2_, *x*_31_), which is the shortest path group most likely to occur, i.e., the one with the minimum shortest path length. The path length is 2.30 and the initial node *x*_2_ (huge ocean wave) of this path is directly connected to the target node *x*_31_ (flooding of decks) without passing through any intermediate nodes.
3. The path_5 (*x*_52_, *x*_31_) and path_14 (*x*_51_, *x*_37_), which are the shortest path groups hardest to block, i.e., the one with the most edges. There are four shortest paths with five edges: the path_5B, the path_5C, the path_14F and the path_14G.

According to the attack effect analysis on different shortest path groups, the attack mode 2 achieves the better effects. In other words, to prevent the occurrence of accidents, it is necessary not only to control the occurrence of risk events (i.e., nodes in the network), but also to block the propagation path between risk events. In addition, due to the limitations imposed by cost, it is impossible to guard against all risk events in the production and operation activities. For the same amount of time and manpower spent, safety investments should be skewed towards risky events that are likely to yield higher safety benefits.

Based on the analysis results of *l*, *GE* and *MK* after attacking different node pairs, the risky events in the following shortest path groups deserve more safety investments: (a) path_3, which consists of eight paths; (b) path_5, which consists of three paths; (c) path_14, which consists of seven paths; (d) path_6, which cconsists of four paths; (e) path_15, which consists of two paths.

In line with the results in Table 4, when some shortest paths are attacked, the connectivity and robustness of the network are greatly reduced. Meanwhile, these paths can also interfere with other shortest paths, indicating that they are critical paths of the entire network. In conclusion, the focus should be on preventing and controlling the following risk events and propagation paths:

1. The path_3E, *x*_3_ (rapid ocean current) → *x*_9_ (collision of vessels) → *x*_23_ (fracture of legs) → *x*_37_ (list of the platform) → *x*_31_ (flooding of decks).
2. The path_5C, *x*_52_ (inadequate risk response) → *x*_53_ (inadequate preventive measures) → *x*_17_ (lack of watertight subdivision isolation) → *x*_33_ (flooding of cabins) → *x*_37_ (list of the platform) → *x*_31_ (flooding of decks).
3. The path_14G, *x*_51_ (inadequate risk perception) → *x*_52_ (inadequate risk response) → *x*_53_ (inadequate preventive measures) → *x*_17_ (lack of watertight subdivision isolation) → *x*_33_ (flooding of cabins) → *x*_37_ (list of the platform).
4. The path_6C, *x*_1_ (strong ocean storm) → *x*_9_ (collision of vessels) → *x*_26_ (fracture of hulls) → *x*_33_ (flooding of cabins).
5. The path_15B, *x*_52_ (inadequate risk response) → *x*_53_ (inadequate preventive measures) → *x*_17_ (lack of watertight subdivision isolation) → *x*_33_ (flooding of cabins) → *x*_37_ (list of the platform).

In the above five shortest paths, three paths all contain *x*_52_ → *x*_53_ → *x*_17_ → *x*_33_ → *x*_37_. It is proved that this shortest path plays an important role in the whole accident evolution network. Therefore, controlling the propagation of this path has great significance for preventing the major storm-disaster-induced accidents in the offshore oil and gas industry.

According to the above analysis results of accident evolution path, different measures are proposed for different stages of accidents. Specific measures are as follows:

1. Preventive measures before accidents: accurate storm forecast and early warning; redundant design of structure and equipment; regular detection of structural defects; fixing movable items on the deck; ensuring the strength of towing ropes; ensuring water-tightness; enhancing risk awareness and strengthening safety training; complete operation regulations and emergency plans; adequate emergency equipment.
2. Control measures in accidents: closing sea valves on the platform; timely drainage of cabin and deck; adjusting ballast conditions of the platform; avoiding excessive oscillation and list of the platform; avoiding hot work; disconnecting from the wellhead; ensuring the normal operation of the power system; ensuring the normal operation of the dynamic positioning system; timely strengthening the support structure; stay out of the storm zone by towing or self-propulsion.
3. Emergency measures after getting out of control: activating the alarm system; emergency muster at the designated location; evacuating platform personnel in sequence; calling for outside help; shutting down unessential electrical equipment; distributing personal survival equipment; lowering lifeboats, life rafts and emergency escape ladders; rescue assist of guard ships.

## 4. Discussion

Compared with the existing literature, on the one hand, the introduction of GT compensates for the shortcomings of the traditional identification of accident causes, which relies too much on the knowledge and experience levels of personnel. GT is characterized by strong objectivity, which can ensure the accuracy and consistency of classification results of accident cause factors. On the other hand, most of the current methods usually divide the causal factors into large categories first and then determine small categories, which leads to the unstable level and content of classification. According to programmatic GT in this paper, a total of 63 concepts, 25 categories, nine main categories, and three core categories were obtained in turn. The result shows that this inductive method can solve the problem well.

Meanwhile, the method of DEMATEL-ISM is improved in this paper. In the establishment of the direct influence matrix, the interaction matrix obtained from the objective analysis in the previous step is introduced based on expert rating. As a result, the strong subjectivity associated with the traditional establishment of the matrix can be avoided. The combination of these two methods retains the advantages of objective induction and subjective reasoning, respectively. 

At the same time, the analysis results of the first two steps provide input for the construction of the CN model, and the three methods form a step-by-step systematic evaluation method. In addition, a hybrid algorithm named HADY is proposed. In previous studies, when calculating the shortest path of CN model, each node pair usually only gets one path. However, the same node pair may have multiple shortest paths. Using HADY, multiple shortest paths under the same node pair can be calculated effectively. We obtained seven shortest paths on the node pair path 14. This is proved that the algorithm is effective and more in line with the actual situation.

Through the actual case study of the proposed method, the importance of different nodes and paths is calculated, and five key nodes and five critical paths are obtained. They are the node *x*_1_ (strong ocean storm), the node *x*_9_ (collision of vessels), the node *x*_31_ (flooding of decks), the node *x*_37_ (list of the platform), the node *x*_51_ (inadequate risk perception), the path_3E (*x*_3_ → *x*_9_ → *x*_23_ → *x*_37_ → *x*_31_), the path_5C (*x*_52_ → *x*_53_ → *x*_17_ → *x*_33_ → *x*_37_ → *x*_31_), the path_14G (*x*_51_ → *x*_52_ → *x*_53_ → *x*_17_ → *x*_33_ → *x*_37_), the path_6C (*x*_1_ → *x*_9_ → *x*_26_ → *x*_33_), and the path_15B (*x*_52_ → *x*_53_ → *x*_17_ → *x*_33_ → *x*_37_). Compared with the research results in the existing literature, the proposed method can not only obtain the importance of nodes, but also obtain the importance of different paths through the method of path attack. The results provide more references for accident prevention.

In general, the proposed method contributes to the precise identification of accident causes and the systematic analysis of accident development. Based on the results of the analysis, reasonable and effective control measures can be implemented to interfere with the development process of accidents. This will slow down or even control the evolution process of accidents under storm disasters to the greatest extent and ensure the safety of offshore oil and gas operations. Further, the application of research results will help reduce casualties caused by major accidents. Meanwhile, it is helpful to achieve sustainable development by reducing the impact of oil and gas accidents on the marine environment.

However, the method proposed in this paper also has its limitations. For example, the method requires detailed data and takes a long time to prepare and code. Moreover, the current CN model of accident evolution cannot realize the dynamic adjustment of nodes and connecting edges. Further research can integrate artificial intelligence in the extraction process of accident causal factors to realize intelligent coding of causal factors for many accident cases. How to embody the dynamic changes of CN models is also one of the important tasks of our future research.

## 5. Conclusions

This paper presents a method for studying the evolution process of accident causes by combining GT, DEMATEL-ISM, and CN. The integrated method covers different stages, including the identification of accident causes, their qualitative analysis, and quantitative assessment, which can be applied for systematically studying the development process of accidents. The application of the proposed method is illustrated by a case study. The results show that inclement weather and lack of risk awareness are the root causes of accidents. Moreover, the damage of support structures and the loss of water-tightness are key nodes of the accident development. Furthermore, the damage of platform facilities is the inevitable path of accident evolution. The constructed accident evolution network model conforms to the characteristics of small-world network and scale-free network. The overall connectivity and robustness of the network are high, and it is difficult to reduce the robustness of the network by attacking a single path or node. Results show that there are five key nodes and five critical paths in the process of accident evolution. Through targeted prevention and control of these nodes and paths, the average shortest path length of the accident evolution network is increased by 35.19%, and the maximum global efficiency decreases by 20.12%. Thus, more attention should be focused on the paths with higher importance as identified by the evaluation results of the attack effect under the limitations imposed by cost, time, and manpower.

## Figures and Tables

**Table 1:** Statistics of coding for causal factors.

Core Category (Coding)	Main Category (Coding)	Category (Coding)	Concept (Coding)	Statistical Frequency
Factors of the external environment (*h*_1_)	Harsh natural environment (*z*_1_)	Inclement weather (*y*_1_)	Strong ocean storm (*x*_1_)	61
			Huge ocean wave (*x*_2_)	61
			Rapid ocean current (*x*_3_)	61
		Adverse factors of sea water (*y*_2_)	Low temperature of sea water (*x*_4_)	5
			Corrosion of sea water (*x*_5_)	4
		Adverse conditions of the seabed (*y*_3_)	Loose soil of the seabed (*x*_6_)	4
	Interference of the objective environment (*z*_2_)	Collision of fixed objects at sea (*y*_4_)	Collision of rocks (*x*_7_)	1
			Collision of buildings (*x*_8_)	1
		Collision of floating objects at sea (*y*_5_)	Collision of vessels (*x*_9_)	2
			Collision of debris (*x*_10_)	2
		Collision of objects on the platform (*y*_6_)	Collision of cantilever deck (*x*_11_)	1
			Collision of cargos (*x*_12_)	2
			Collision of lifeboats (*x*_13_)	1
Factors of structure and equipment (*h*_2_)	Defect of structure design (*z*_3_)	Defect of structural connection (*y*_7_)	Poor quality of welded joints (*x*_14_)	2
			Improper screws (*x*_15_)	1
		Defect of watertight facility (*y*_8_)	Lack of weather seal (*x*_16_)	1
			Lack of watertight subdivision isolation (*x*_17_)	1
		Defect of dimension and material (*y*_9_)	Weak strength of hulls (*x*_18_)	1
			Design defect of single hull tankers (*x*_19_)	1
			Insufficient height of decks (*x*_20_)	3
	Damage to platform structure (*z*_4_)	Damage of support structures (*y*_10_)	Fatigue propagation of weld cracks (*x*_21_)	3
			Fracture of lateral braces (*x*_22_)	2
			Fracture of legs (*x*_23_)	10
			Fracture of pontoons (*x*_24_)	1
		Damage of watertight structures (*y*_11_)	Fracture of portholes (*x*_25_)	4
			Fracture of hulls (*x*_26_)	7
		Damage of platform facilities (*y*_12_)	Collapse of derricks (*x*_27_)	7
			Damage of dormitories (*x*_28_)	2
			Fracture of pipelines (*x*_29_)	3
			Damage of helidecks (*x*_30_)	2
	Unbalance state of the platform (*z*_5_)	Loss of watertightness (*y*_13_)	Flooding of decks (*x*_31_)	21
			Flooding of ballast control rooms (*x*_32_)	2
			Flooding of cabins (*x*_33_)	12
		Poor state of the platform (*y*_14_)	Excessive oscillation of legs (*x*_34_)	2
			High center of gravity (*x*_35_)	1
			Oscillation of the platform (*x*_36_)	3
			List of the platform (*x*_37_)	12
	Failure of platform equipment (*z*_6_)	Failure of the dynamic positioning system (*y*_15_)	Failure of thrusters (*x*_38_)	1
			Breaking of mooring lines (*x*_39_)	2
			Breaking of towing lines (*x*_40_)	5
		Failure of the power system (*y*_16_)	Failure of the power system (*x*_41_)	3
		Failure of emergency equipment (*y*_17_)	Damage of lifeboats (*x*_42_)	2
			Sinking of lifeboat (*x*_43_)	2
			Poor performance of protective clothing (*x*_44_)	1
		Failure of communication equipment (*y*_18_)	Failure of radio equipment (*x*_45_)	1
Factors of humans and management (*h*_3_)	Inappropriate personnel operation (*z*_7_)	Violation operation (*y*_19_)	Violation of hot work (*x*_46_)	2
			Violation of towing operation (*x*_47_)	2
			Wrong wellhead connection (*x*_48_)	1
		Weak fixation of equipment (*y*_20_)	Weak fixation of cargos (*x*_49_)	2
			Weak fixation of lifeboats (*x*_50_)	1
	Lack of risk management (*z*_8_)	Lack of risk awareness (*y*_21_)	Inadequate risk perception (*x*_51_)	12
			Inadequate risk response (*x*_52_)	12
		Inadequate safety training (*y*_22_)	Inadequate preventive measures (*x*_53_)	1
			Inadequate accident treatment experience (*x*_54_)	5
	Inadequate emergency management (*z*_9_)	Insufficient emergency equipment (*y*_23_)	Insufficient auxiliary vessels (*x*_55_)	1
			Insufficient lifeboats (*x*_56_)	1
			Insufficient emergency protective equipment (*x*_57_)	1
		Incomplete rules and regulations (*y*_24_)	Incomplete operation procedures (*x*_58_)	2
			Incomplete emergency plans (*x*_59_)	1
		Inappropriate emergency operation (*y*_25_)	Inappropriate emergency decision making (*x*_60_)	3
			Inappropriate emergency commands (*x*_61_)	1
			Inappropriate emergency measures (*x*_62_)	3
			Inappropriate emergency rescues (*x*_63_)	3

**Table 2:** Results of *CC_i_* and *BC_i_*.

Node	*CC_i_*/10^−1^	*BC_i_*	Node	*CC_i_*/10^−1^	*BC_i_*	Node	*CC_i_*/10^−1^	*BC_i_*	Node	*CC_i_*/10^−1^	*BC_i_*
*x*_1_	1.98	0.00	*x*_17_	0.83	38.54	*x*_33_	1.25	180.44	*x*_49_	3.00	18.13
*x*_2_	1.78	6.00	*x*_18_	1.67	28.00	*x*_34_	1.19	35.67	*x*_50_	3.00	15.25
*x*_3_	3.45	0.00	*x*_19_	5.00	0.00	*x*_35_	1.67	6.67	*x*_51_	0.83	0.00
*x*_4_	2.50	0.00	*x*_20_	0.00	2.12	*x*_36_	3.01	37.33	*x*_52_	1.67	12.96
*x*_5_	0.44	0.00	*x*_21_	2.45	8.00	*x*_37_	2.24	246.74	*x*_53_	1.48	101.11
*x*_6_	0.00	0.00	*x*_22_	2.27	0.00	*x*_38_	0.50	261.10	*x*_54_	2.00	0.00
*x*_7_	1.81	75.72	*x*_23_	2.42	93.12	*x*_39_	3.00	6.83	*x*_55_	4.50	0.92
*x*_8_	1.57	85.52	*x*_24_	2.36	13.35	*x*_40_	2.22	17.65	*x*_56_	4.50	0.92
*x*_9_	1.58	131.13	*x*_25_	2.62	59.22	*x*_41_	1.25	356.00	*x*_57_	4.50	0.92
*x*_10_	2.03	47.59	*x*_26_	2.00	75.23	*x*_42_	2.32	38.25	*x*_58_	1.67	22.00
*x*_11_	2.48	16.81	*x*_27_	2.09	8.45	*x*_43_	1.19	0.00	*x*_59_	3.00	2.00
*x*_12_	2.21	114.51	*x*_28_	2.00	0.00	*x*_44_	5.00	0.00	*x*_60_	3.67	0.00
*x*_13_	2.28	100.28	*x*_29_	2.08	0.00	*x*_45_	0.00	55.90	*x*_61_	3.67	0.00
*x*_14_	2.50	3.14	*x*_30_	2.08	0.00	*x*_46_	1.67	2.58	*x*_62_	2.86	1.50
*x*_15_	5.00	0.00	*x*_31_	1.21	213.13	*x*_47_	1.00	34.92	*x*_63_	2.33	5.25
*x*_16_	1.00	62.54	*x*_32_	1.43	112.70	*x*_48_	5.00	0.00			

**Table 3:** Shortest paths of accident evolution.

No.	Code	Shortest Path	Length	No.	Code	Shortest Path	Length
1	path_1	*x*_1_ → *x*_2_ → *x*_31_	4.61	23	path_9A	*x*_51_ → *x*_17_ → *x*_33_	9.21
2	path_2	*x*_2_ → *x*_31_	2.30	24	path_9B	*x*_51_ → *x*_53_ → *x*_17_ → *x*_33_	
3	path_3A	*x*_3_ → *x*_9_ → *x*_24_ → *x*_31_	13.82	25	path_9C	*x*_51_ → *x*_52_ → *x*_53_ → *x*_17_ → *x*_33_	
4	path_3B	*x*_3_ → *x*_9_ → *x*_37_ → *x*_31_		26	path_10	*x*_52_ → *x*_53_ → *x*_17_ → *x*_33_	6.91
5	path_3C	*x*_3_ → *x*_10_ → *x*_24_ → *x*_31_		27	path_11	*x*_1_ → *x*_37_	
6	path_3D	*x*_3_ → *x*_11_ → *x*_37_ → *x*_31_		28	path_12	*x*_2_ → *x*_37_	
7	path_3E	*x*_3_ → *x*_9_ → *x*_23_ → *x*_37_ → *x*_31_		29	path_13A	*x*_3_ → *x*_9_ → *x*_37_	11.51
8	path_3F	*x*_3_ → *x*_9_ → *x*_24_ → *x*_37_ → *x*_31_		30	path_13B	*x*_3_ → *x*_11_ → *x*_37_	
9	path_3G	*x*_3_ → *x*_10_ → *x*_23_ → *x*_37_ → *x*_31_		31	path_13C	*x*_3_ → *x*_9_ → *x*_23_ → *x*_37_	
10	path_3H	*x*_3_ → *x*_10_ → *x*_24_ → *x*_37_ → *x*_31_		32	path_13D	*x*_3_ → *x*_9_ → *x*_24_ → *x*_37_	
11	path_4	*x*_51_ → *x*_20_ → *x*_31_	6.91	33	path_13E	*x*_3_ → *x*_10_ → *x*_23_ → *x*_37_	
12	path_5A	*x*_52_ → *x*_58_ → *x*_47_ → *x*_31_	11.51	34	path_13F	*x*_3_ → *x*_10_ → *x*_24_ → *x*_37_	
13	path_5B	*x*_52_ → *x*_53_ → *x*_16_ → *x*_32_ → *x*_37_ → *x*_31_		35	path_14A	*x*_51_ → *x*_14_ → *x*_23_ → *x*_37_	11.51
14	path_5C	*x*_52_ → *x*_53_ → *x*_17_ → *x*_33_ → *x*_37_ → *x*_31_		36	path_14B	*x*_51_ → *x*_16_ → *x*_32_ → *x*_37_	
15	path_6A	*x*_1_ → *x*_2_ → *x*_33_	9.21	37	path_14C	*x*_51_ → *x*_17_ → *x*_33_ → *x*_37_	
16	path_6B	*x*_1_ → *x*_2_ → *x*_31_ → *x*_33_		38	path_14D	*x*_51_ → *x*_53_ → *x*_16_ → *x*_32_ → *x*_37_	
17	path_6C	*x*_1_ → *x*_9_ → *x*_26_ → *x*_33_		39	path_14E	*x*_51_ → *x*_53_ → *x*_17_ → *x*_33_ → *x*_37_	
18	path_6D	*x*_1_ → *x*_10_ → *x*_26_ → *x*_33_		40	path_14F	*x*_51_ → *x*_52_ → *x*_53_ → *x*_16_ → *x*_32_ → *x*_37_	
19	path_7A	*x*_2_ → *x*_33_	6.91	41	path_14G	*x*_51_ → *x*_52_ → *x*_53_ → *x*_17_ → *x*_33_ → *x*_37_	
20	path_7B	*x*_2_ → *x*_31_ → *x*_33_		42 43	path_15A path_15B	*x*_52_ → *x*_53_ → *x*_16_ → *x*_32_ → *x*_37_ *x*_52_ → *x*_53_ → *x*_17_ → *x*_33_ → *x*_37_	9.21
21	path_8A	*x*_3_ → *x*_9_ → *x*_26_ → *x*_33_	11.51				
22	path_8B	*x*_3_ → *x*_10_ → *x*_26_ → *x*_33_					

**Table 4:** Importance of different shortest paths in the same group.

Code	*GE*/10^−2^	*MK*	*MS*	*PS*	Sorting	Code	*GE*/10^−2^	*MK*	*MS*	*PS*	Sorting
path_3A	3.80	18	5	0.76	6	path_9A	4.03	21	2	2.00	3
path_3B	3.60	18	6	1.57	5	path_9B	3.76	21	2	2.80	2
path_3C	3.88	19	4	0.00	8	path_9C	3.69	21	2	3.00	1
path_3D	3.70	19	5	0.75	7	path_13A	3.85	19	2	1.70	4
path_3E	3.45	15	6	2.67	1	path_13B	3.95	20	0	0.00	6
path_3F	3.49	16	7	2.65	2	path_13C	3.77	18	3	3.00	1
path_3G	3.53	16	6	2.24	3	path_13D	3.80	18	3	2.81	2
path_3H	3.57	17	7	2.23	4	path_13E	3.84	19	2	1.75	3
path_5A	3.90	21	0	0.00	3	path_13F	3.88	19	2	1.56	5
path_5B	3.45	19	1	2.51	2	path_14A	4.00	20	0	0.00	7
path_5C	3.37	18	1	3.00	1	path_14B	3.93	20	2	0.63	6
path_6A	3.75	20	1	2.09	3	path_14C	3.84	19	2	1.81	4
path_6B	3.97	21	1	1.00	4	path_14D	3.65	20	4	1.67	5
path_6C	3.68	18	1	3.00	1	path_14E	3.56	19	4	2.85	2
path_6D	3.75	19	1	2.42	2	path_14F	3.58	20	4	1.81	3
path_7A	3.79	20	0	3.00	1	path_14G	3.48	19	4	3.00	1
path_7B	4.01	21	0	1.00	2	path_15A	3.65	20	1	1.00	2
path_8A	3.68	18	1	3.00	1	path_15B	3.80	19	1	3.00	1
path_8B	3.75	19	1	1.00	2						

**Figure A1:**
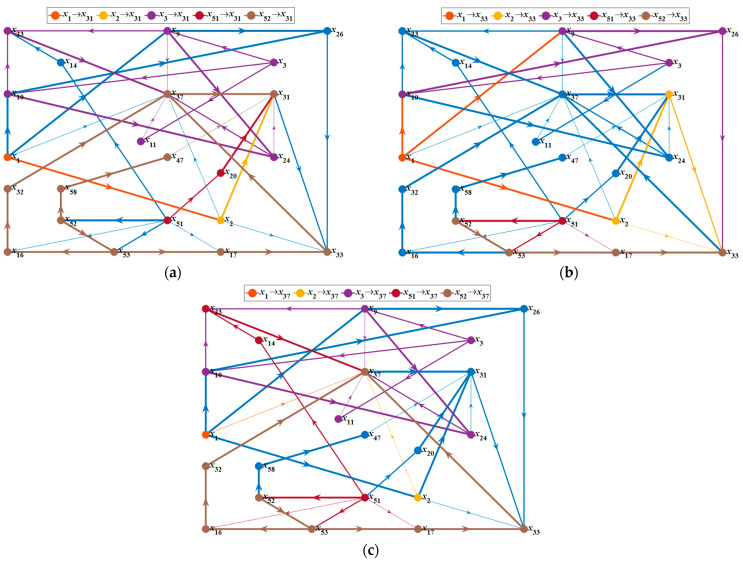
Network diagram of shortest paths targets (**a**) *x*_31_, (**b**) *x*_33_ and (**c**) *x*_37_.

## Data Availability

Not applicable.

## Abbreviations

GT, Grounded theory; DEMATEL, Decision making trial and evaluation laboratory; ISM, Interpretative structural modelling; CN, Complex networks; RCA, Root cause analysis; FTA, Fault tree analysis; ETA, Event tree analysis; AHP, Analytic hierarchy process; ANP, Analytic network process; SEM, Structural equation modelling; BN, Bayesian network; HADY, Hybrid algorithm of Dijkstra and Yen; GOM, Gulf of Mexico; UKCS, United Kingdom continental shelf; NCS, Norwegian continental shelf; WP, Western Pacific.

## Annotations

*X*—the set of concepts; *Y*—the set of categories; ***F***—the direct influence matrix; ***C***—the normal matrix; ***T***—the comprehensive influence matrix; ***I***—the identity matrix; ***H***—the overall influence matrix; ***K***—the reachable matrix; λ—the threshold value; *b_i_*—the influencing degree; *d_i_*—the influenced degree; *c_i_*—the centrality; *a_i_*—the causality; *Q_i_*—the antecedent set; *P_i_*—the reachable set; *m_i_*—the factor degree; ***S***—the direct interaction matrix; ***R***—the interaction matrix of categories; ***RX***—the interaction matrix of concepts; ***A***—the adjacency matrix; *G*—the CN graph model; *V*—the set of nodes; *E*—the set of edges; *W*—the set of weights; *k_i_*—the node degree; $k_{i}^{\mathrm{out}}$—the out-degree; $k_{i}^{\mathrm{in}}$— the in-degree; *P*(*k*)—the degree distribution; *CC_i_*—the clustering coefficient; *CC*—the network clustering coefficient; *BC_i_*—the betweenness centrality; $BC_{i}^{\prime }$—the normalized betweenness centrality; *l_ij_*—the shortest path length; *D*—the network diameter; *L*—the average shortest path length; *GE*—the global efficiency; *MK*—the maximum strongly connected component scale; *ew_ij_*—the entropy weight.

## Appendix A

**Table A1:** Statistics of major accidents in the offshore oil and gas industry.

No.	Region	Platform Type	Consequence	No.	Region	Platform Type	Consequence
1	GOM	Jack up	Sinking	40	WP	Vessel	Sinking
2	UKCS	Semi-sub	Sinking	41	GOM	Vessel	Major damage
3	GOM	Jack up	Sinking	42	GOM	Jack up	Major damage
4	GOM	Jack up	Sinking	43	GOM	Spar	Major damage
5	UKCS	Jack up	Sinking	44	GOM	Jacket	Collapse
6	UKCS	Semi-sub	Capsizing	45	GOM	Semi-sub	Major damage
7	GOM	Jack up	Sinking	46	WP	Jacket	Collapse
8	NCS	Jack up	Sinking	47	GOM	Semi-sub	Major damage
9	NCS	Semi-sub	Capsizing	48	GOM	Tension leg	Major damage
10	GOM	Jack up	Capsizing	49	GOM	Jack up	Major damage
11	WP	Jack up	Sinking	50	GOM	Jack up	Major damage
12	NCS	Jack up	Sinking	51	GOM	Jack up	Sinking
13	WP	Jack up	Major damage	52	GOM	Semi-sub	Major damage
14	GOM	Jack up	Sinking	53	GOM	Jacket	Collapse
15	NCS	Semi-sub	Capsizing	54	GOM	Jack up	Major damage
16	GOM	Jack up	Sinking	55	GOM	Tension leg	Major damage
17	NCS	Jack up	Sinking	56	GOM	Jack up	Collapse
18	NCS	Jack up	Sinking	57	GOM	Jack up	Collapse
19	NCS	Jack up	Sinking	58	GOM	Jacket	Major damage
20	GOM	Semi-sub	Capsizing	59	GOM	Jack up	Sinking
21	NCS	Jack up	Capsizing	60	GOM	Jack up	Sinking
22	GOM	Jack up	Major damage	61	GOM	Jack up	Major damage
23	WP	Vessel	Capsizing	62	GOM	Jack up	Sinking
24	NCS	Jack up	Sinking	63	GOM	Jacket	Collapse
25	GOM	Jack up	Capsizing	64	GOM	Jacket	Major damage
26	GOM	Jack up	Sinking	65	GOM	Semi-sub	Major damage
27	WP	Vessel	Capsizing	66	GOM	Vessel	Major damage
28	UKCS	Jack up	Sinking	67	WP	Vessel	Major damage
29	UKCS	Jack up	Capsizing	68	WP	Vessel	Major damage
30	WP	Vessel	Capsizing	69	GOM	Semi-sub	Major damage
31	GOM	Jack up	Major damage	70	GOM	Vessel	Capsizing
32	NCS	Jack up	Sinking	71	GOM	Jack up	Major damage
33	NCS	Jack up	Sinking	72	GOM	Jacket	Collapse
34	GOM	Jack up	Sinking	73	GOM	Jacket	Collapse
35	GOM	Jack up	Capsizing	74	WP	Semi-sub	Major damage
36	GOM	Jack up	Capsizing	75	WP	Jack up	Major damage
37	GOM	Jack up	Capsizing	76	WP	Jack up	Capsizing
38	GOM	Jacket	Major damage	77	WP	Jacket	Major damage
39	GOM	Jacket	Collapse	78	WP	Semi-sub	Capsizing
